# Arabidopsis TAF1 is an MRE11‐interacting protein required for resistance to genotoxic stress and viability of the male gametophyte

**DOI:** 10.1111/tpj.13020

**Published:** 2015-10-08

**Authors:** Wanda M. Waterworth, Georgina E. Drury, George Blundell‐Hunter, Christopher E. West

**Affiliations:** ^1^ Centre for Plant SciencesUniversity of LeedsWoodhouse LaneLeedsLS2 9JTUK; ^2^ Present address: Medical Research Council1 Kemble StreetLondonWC2B 4ANUK; ^3^ Present address: School of Life SciencesQueen's Medical CentreUniversity of NottinghamNottinghamNG7 2UHUK

**Keywords:** DNA repair, recombination, transcription, DNA double‐strand break, *Arabidopsis thaliana*

## Abstract

Repair of DNA double‐strand breaks (DSBs) by recombination pathways is essential for plant growth and fertility. The recombination endonuclease MRE11 plays important roles in sensing and repair of DNA DSBs. Here we demonstrate protein interaction between Arabidopsis MRE11 and the histone acetyltransferase TAF1, a TATA‐binding protein Associated Factor (TAF) of the RNA polymerase II transcription initiation factor complex TFIID. Arabidopsis has two TAF1 homologues termed TAF1 and TAF1b and mutant *taf1b* lines are viable and fertile. In contrast, *taf1* null mutations are lethal, demonstrating that *TAF1* is an essential gene. Heterozygous *taf1* ^*+/−*^ plants display abnormal segregation of the mutant allele resulting from defects in pollen tube development, indicating that TAF1 is important for gamete viability. Characterization of an allelic series of *taf1* lines revealed that hypomorphic mutants are viable but display developmental defects and reduced plant fertility. Hypersensitivity of *taf1* mutants lacking the C‐terminal bromodomain to X‐rays and mitomycin C, but not to other forms of abiotic stress, established a specific role for TAF1 in plant DNA repair processes. Collectively these studies reveal a function for TAF1 in plant resistance to genotoxic stress, providing further insight into the molecular mechanisms of the DNA damage response in plants.

## Significance Statement

DNA recombination pathways are critical for genome stability, fertility and survival of organisms. Here we establish a molecular link between a recombination endonuclease and a histone acetyltransferase involved in transcription initiation, providing insight into chromosomal break detection and DNA repair processes.

## Introduction

DNA repair, recombination and replication all take place in the context of chromatin structure. Packaging of DNA into chromatin can modulate access of DNA damage detection and repair complexes. Chromatin structure can be altered by chromatin remodelling enzymes or covalent modification including phosphorylation and acetylation. Histone acetylation/deacetylation plays key roles in regulation of transcriptional regulation, whilst modification of lysine residues in the histone tails of H3 and H4 is important to recombinational repair of chromosomal breaks in yeast and mammals (Bird *et al*., 2002; Tamburini and Tyler, 2005). Acetylation or deacetylation of specific lysine residues in histone proteins is mediated by transcription cofactors which are often associated with histone acetyltransferase (HAT) and histone deacetylation (HDAC) activities. In plants, HATs are classified into three families that are also present in other eukaryotes. These include the p300/CREB binding protein family, the TAF1 family and the GNAT (GCN5‐related N‐terminal acetyltransferases)‐MYST superfamily (Pandey *et al*., 2002). HAT‐mediated histone acetylation has diverse roles in transcriptional regulation in plant development and in response to environmental stimuli.

Proteins of the TAF1 family participate in transcription initiation as part of the RNA polymerase II pre‐initiation complex, comprised of RNA polymerase II and a subset of core transcription factors (TFIIA, B, D, E, F and H). Initial promoter recognition is mediated by the basal transcription factor TFIID, which includes the TATA‐binding protein (TBP) in a complex with several evolutionary conserved TBP‐associated factors (TAFs). TAFs function in basal transcription and can act as transcriptional coactivators in transcriptional complex assembly and in promoter recognition. TAF1 (also termed TAF_II_250) is conserved across eukaryotes and is essential for TFIID function (Wassarman and Sauer, 2001). TAF1 possesses several enzymatic activities including protein kinase activity (Maile *et al*., 2004), ubiquitination (Pham and Sauer, 2000) and histone acetyltransferase activities (Mizzen *et al*., 1996). C‐terminal bromodomains target TAF1 to acetylated histones H4, H3 and H2A *in vivo* and TAF1 acetyltransferase activity results in further histone acetylation, promoting transcription (Martinez, 2002; Kanno *et al*., 2004).

Arabidopsis has two TAF1 related genes, termed HAF1 (At1g32750) and HAF2 (At3g19040) (Pandey *et al*., 2002) or TAF1 and TAF1b (Lago *et al*., 2004). The TAF1N‐terminal domain (TAND) was shown to be important for interaction with TBP in yeast and Drosophila (Lawit *et al*., 2007). The TAND domain is present in TAF1 in Arabidopsis, rice and Drosophila, but is mostly absent in the TAF1b isoform (Lawit *et al*., 2007) which has specific developmental roles, mediating light‐induced responses. Mutant *haf2* plants are viable but produce lighter coloured seedlings with a reduced chlorophyll content than wild‐type plants (Bertrand *et al*., 2005). Transcriptional responses are mediated by HAF2/TAF1b acetylation of histone H3 and H4 in the promoter of the light‐induced genes, including the gene encoding small subunit of RUBISCO (*RBS‐1A*) (Benhamed *et al*., 2006). In contrast, no major growth defects were identified in the *haf1* (*taf1‐3*) line investigated in the study of Bertrand *et al*. (2005)*,* which supported the hypothesis that the essential core transcriptional functions are shared redundantly between HAF1 and HAF2 (TAF1/TAF1b).

Chromatin modification by acetylation and HAT activities have important roles in DSB repair in mammals and yeast (Bird *et al*., 2002; Tamburini and Tyler, 2005). However, early events in eukaryotic DNA damage detection and repair remain to be elucidated in plants. Recent work has additionally identified that changes in histone acetylation patterns are associated with the plant DNA damage response (Raut and Sainis, 2011; Drury *et al*., 2012). Here we establish interaction between the histone acetyltransferase TAF1 with MRE11, a factor involved in DNA DSB repair. MRE11 is a core component of the conserved MRE11‐RAD50‐NBS1 (MRN) complex which plays important roles in DNA double‐strand break (DSB) detection and repair in both vegetative and meiotic tissues (Bundock and Hooykaas, 2002; Puizina *et al*., 2004; Waterworth *et al*., 2011). Interaction was identified by yeast two‐hybrid library screening and confirmation *in vivo* further localised the site MRE11 interaction to the bromodomain of TAF1. Further characterisation of TAF1 identified that this transcription factor, unlike TAF1b, is essential for plant viability and fertility. Specific hypersensitivity of *taf1* mutant lines to genotoxins that induce DNA DSBs and interstrand DNA crosslinks, but not other forms of abiotic stress, demonstrates a role for TAF1 in plant responses to DSBs. This study reveals a molecular link between basal transcription and recombination factors and establishes a requirement for TAF1 in genotoxic stress resistance in plants.

## Results

### TAF1 interacts with the recombination endonuclease MRE11

**Figure 1:**
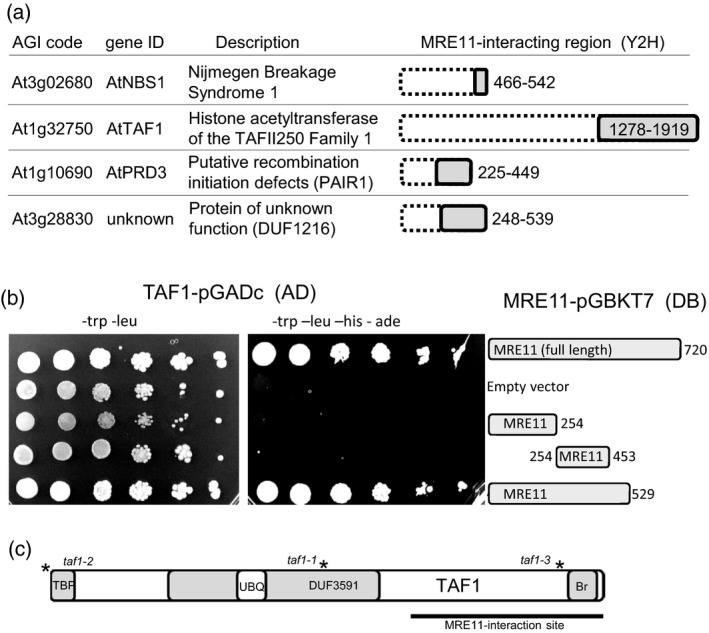
Yeast two‐hybrid analysis identifies the interaction between MRE11 and TAF1. (a) MRE1‐interacting proteins isolated in the yeast two‐hybrid screen. Proteins identified showing the regions isolated in the Y2H screen. (b) Confirmation of the yeast two‐hybrid analysis shows the interaction between MRE11 and TAF1 requires the central region of MRE11 and the C‐terminal region of TAF1. (c) TAF1 schematic showing an N‐terminal TATA‐binding protein interaction domain (TBP), a conserved HAF1 domain (DUF5391), a ubiquitin domain (UBQ) and a C‐terminal bromodomain (Br). Asterisks correspond to positions of T‐DNA insertions in the *TAF1* coding sequence.

MRE11 has endo‐ and exonuclease activities and plays a central role in eukaryotic DNA repair and DNA damage signalling. The endonuclease activity is essential for meiotic recombination, and *mre11* mutant lines also display defects in DSB repair in vegetative cells (Heacock *et al*., 2004). MRE11 was previously shown to interact with NBS1 and RAD50 in plants, forming the conserved eukaryotic MRN complex (Waterworth *et al*., 2007). RAD50 has structural roles in bridging DNA ends, whereas NBS1 in mammals has intracellular signalling activity, mediated in part through interaction with the protein kinase ATM (Falck *et al*., 2005). Yeast two‐hybrid library screening for proteins that interact with Arabidopsis MRE11 confirmed the previously identified interaction between NBS1 and MRE11 (Waterworth *et al*., 2007) (Figure 1a), isolating an NBS1 fragment that included the conserved ‘FKRFKR’ MRE11‐interacting motif (residues 467–472 of NBS1). In addition three interactions were identified, including the meiotic protein AtPRD3, required for DSB formation in early meiosis (De Muyt *et al*., 2009), and an uncharacterised protein (At3g28830). Specific interaction was also identified between the core transcription factor TAF1 and MRE11, mediated by the C‐terminal third of TAF1 in a region distal to the histone acetyltransferase catalytic domain but containing a bromodomain, a conserved protein motif involved in binding acetylated lysine residues. A series of MRE11 deletion constructs was tested to further delineate the region of MRE11 that mediates interaction with TAF1 (Figure 1b), and revealed that the N‐terminal and middle region of MRE11 are not sufficient for interaction, that the C‐terminal domain is not required, but that MRE11 up to residue 529 is required for the interaction with TAF1. This interaction prompted further investigation into the roles of TAF1 in plants.

### Arabidopsis contains two TAF1‐related genes

*TAF1* (AT1G32750) is a 10.5 kbp gene encoding a 1919 aa protein (Pandey *et al*., 2002) with 27.5% similarity to human TAF1. Highest sequence conservation is localised to domains including the TBP‐binding region, the histone acetyltransferase region, the bromodomain and ubiquitin associated domains (Figure 1c). TAF1b (AT3G19040, HAF2), previously implicated in control of light regulated developmental processes in plants, displays 24.9% similarity with hTAF1 (Earley *et al*., 2007) and lacks the N‐terminal 90 aa containing the TBP‐domain found in AtTAF1 and hTAF1 (Lawit *et al*., 2007). TAF1b (HAF2) is involved in transcriptional responses to light, but analysis of TAF1 has not previously been reported in detail. To investigate the function of TAF1 in Arabidopsis, two independent T‐DNA insertion mutants were obtained from the SALK collection (Alonso *et al*., 2003) and a third mutant line was isolated from the SAIL collection (Sessions *et al*., 2002) (Figure 1c).

### TAF1 is an essential gene

**Figure 2:**
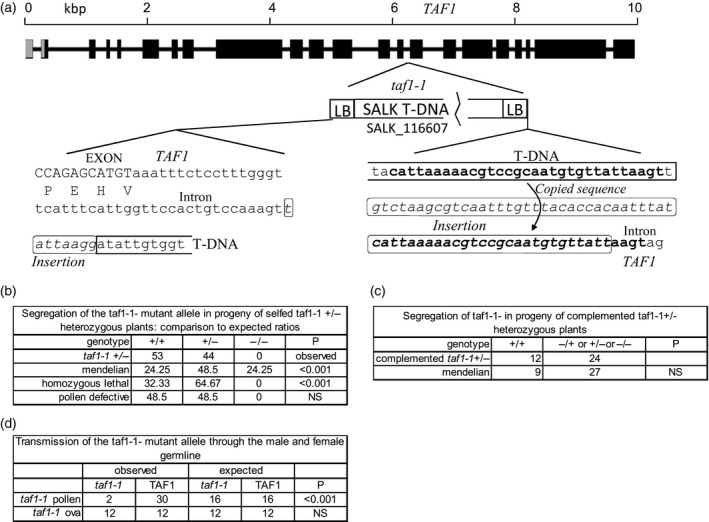
Segregation analysis of the *taf1‐1* mutant allele. (a) Schematic of the TAF1 gene showing the positions of T‐DNA insertion in the *taf1‐1* line. Exons are shown as black boxes, introns as lines and untranslated regions are represented as grey boxes. (b) Analysis of the segregation of the *taf1‐1* allele. Chi‐squared analysis (*P*) of segregation ratios indicates that *taf1‐1* is consistent with loss in transmission through the male or female line. NS = not significant. (c) Segregation of the *taf1‐1* mutant allele in complemented *taf1‐1* heterozygous lines is not significantly different to wild‐type. (d) Segregation of the *taf1‐1* allele is normal through the female line, while the *taf1‐1* allele displays greatly reduced transmission through pollen.

The *taf1‐1* mutant line (SALK_116607) contained an insertion site localised to the intron between exons 14 and 15 (Figure 2a), corresponding to the highly conserved histone acetyltransferase catalytic domain shared amongst TAF1 homologues (Figure 1c). Despite extensive genotyping (*n* > 900), no plants homozygous for the *taf1‐1* allele were obtained, suggesting that *TAF1* is an essential gene in Arabidopsis. The requirement for TAF1 was confirmed by the observation that homozygous *taf1‐1* plants were only viable when complemented by the wild‐type gene (Figure 2b). This indicates that *TAF1* is an essential gene in Arabidopsis and is not redundant to *TAF1b*. This suggests that during plant evolution, *TAF1* gene duplication led to specialisation of the TAF1b isoform in control of light regulated gene expression and that TAF1 retained the core transcriptional activities.

### TAF1 is important for viability of the male gametophyte

PCR genotyping of the progeny of a selfed *taf1‐1* ^*+/−*^ hemizygous line revealed non‐Mendelian segregation of the mutant allele, with the *taf1‐1* ^*−*^ allele significantly underrepresented in the segregating population (Figure 2b). Progeny from the selfed hemizygote differed significantly from both normal segregation (1:2:1 of homozygous mutants:heterozygous plants:wild‐type plants) and also from the 2:1 segregation predicted for recessive lethal mutant alleles (*P* < 0.01, chi‐squared test, *n* = 97, Figure 2b). The ratio of wild‐type:hemizygous plants was not significantly different to the 1:1 ratio which would result from expected for loss of the mutant allele through either the male or female germline (*P* > 0.05). Complementation with wild‐type *TAF1* gene restored segregation of the mutant allele to Mendelian ratios (*P* > 0.05, chi‐squared test, *n* = 36) and rescued the abnormal segregation seen in the heterozygous mutant lines (*P* < 0.01, Figure 2c). Outcrossing from hemizygous *taf1‐1* ^*+/−*^ plants indicated a strong defect in transmission of the *taf1‐1* allele through the male line, accounting for only ~6% crossed progeny, significantly different from the 50% normal transmission (*P* < 0.01, chi‐squared test, *n* = 36, Figure 2d). The *taf1‐1* mutant allele showed normal inheritance through the female line, with half the progeny carrying the allele when mutant plants were cross‐fertilised with wild‐type pollen (*P* > 0.05, chi‐squared test, *n* = 24, Figure 2d).

### Loss of the *taf1‐1* allele results from impaired pollen tube growth

**Figure 3:**
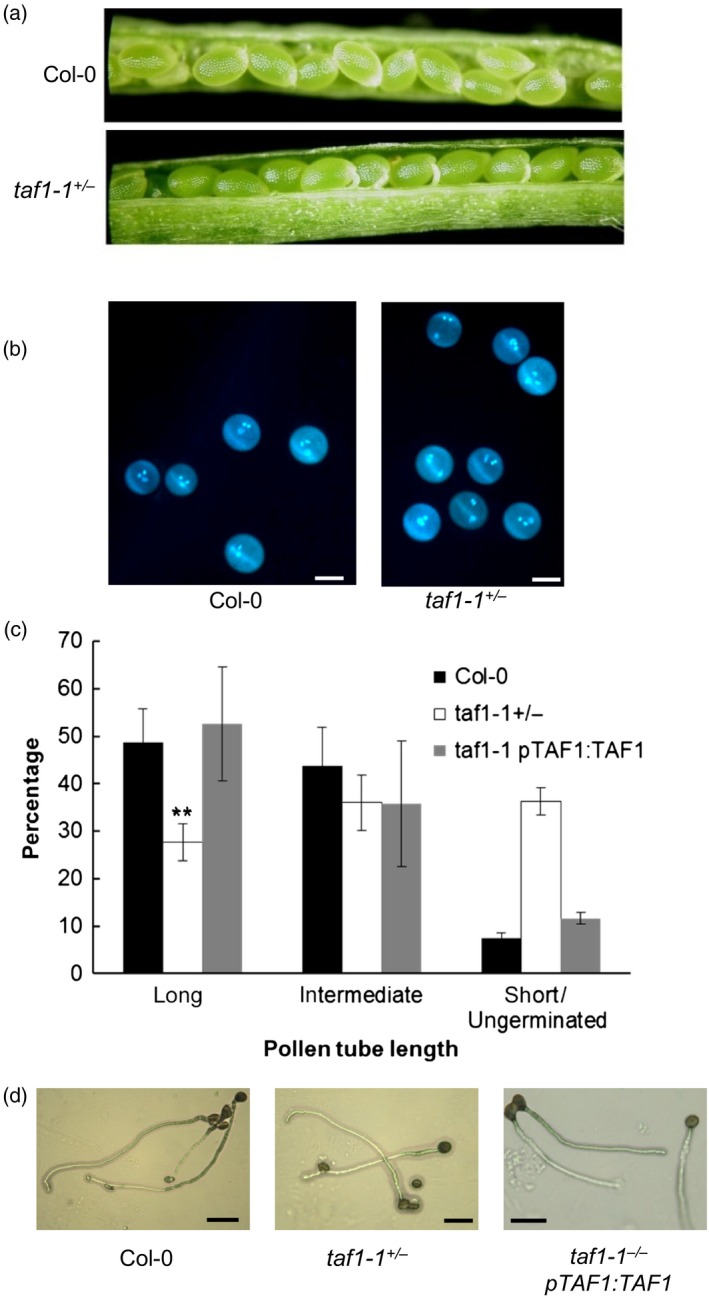
Abnormal *taf1‐1* segregation results from defects in pollen tube growth. (a) *taf1‐1* hemizygotes display no increased incidence of aborted seeds. (b) Pollen development is normal in *taf1‐1* mutant lines, with tricellular pollen evidence in DAPI‐staining of wild‐type and *taf1‐1* hemizygotes. Scale bar represents 20 μm. (c) Pollen tube growth is defective in *taf1‐1* hemizygotes, which display approximately half the number of full‐length pollen tubes relative to wild‐type lines. Error bars represent the SEM of 3 or more replicates of > 10 pollen grains. (d) Complementation with the wild‐type *TAF1* restores pollen tube growth to wild‐type levels. Bar represents 50 μm.

Loss of mutant alleles through the male line can arise as a consequence of defects in gametophyte development. Wild‐type pollen undergoes two rounds of cell division on completion of meiosis, initially to produce the generative and vegetative nuclei, whilst subsequent division of the generative cell results in mature, tricellular pollen. Failure to complete these divisions results in immature, non‐viable pollen and loss of the mutant allele. Bicellular pollen (containing one generative cell) results in fertilisation of the ovule but not the endosperm, resulting in a characteristic early abortion in early embryogenesis. Inspection of *taf1‐1* ^*+/−*^ siliques found no evidence of increased embryo abortion relative to wild‐type plants (Figure 3a). In support of this observation, fluorescence microscopy of DAPI‐stained pollen indicated no increase in the incidence of immature pollen in *taf1‐1* ^*+/−*^ plants, with high levels of tricellular pollen with two generative nuclei clearly visible (Figure 3b). Therefore, the *taf1‐1* mutation did not affect pollen grain development, indicative that downstream processes may be impaired in the mutant background.

Analysis of pollen tube growth in heterozygous *taf1‐1* ^*+/−*^ mutants showed a significant decrease in the number of full‐length pollen tubes relative to wild‐type lines (Figure 3c,d), consistent with the reduced transmission of the *taf1‐1* ^*−*^ allele. In heterozygous *taf1‐1* ^*+/−*^ mutants around 50% mature pollen grains failed to germinate or displayed poor growth compared to both Col‐0 controls and complemented *taf1‐1* mutant lines carrying the wild‐type *TAF1* gene. Taken together, these results indicate that TAF1 is essential for plant viability and loss of TAF1 in haploid pollen severely reduces, although does not abolish, pollen viability.

### T‐DNA insertion upstream of TAF1 results in ectopic expression of a mutant transcript in *taf1‐2* mutant lines

**Figure 4:**
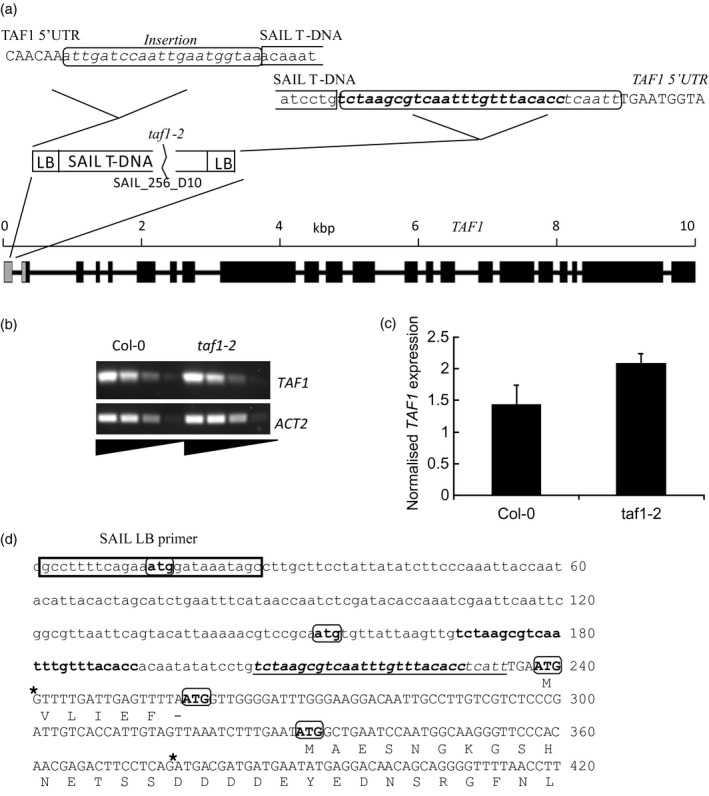
*taf1‐2* mutants express a mutant *TAF1* transcript. (a) *taf1‐2* contains a SAIL T‐DNA inserted in the *TAF1* 5′ untranslated region. The *TAF1* 5′ UTR is shown in uppercase, introns as lower case and inserted sequence in italics. Analysis of left border sequences is consistent with a head‐to‐head double insertion of the T‐DNA. (b) Comparison of *TAF1* expression levels in Col‐0 and *taf1‐2* mutants by semi‐quantitative PCR. (c) Q‐PCR normalised to *ACTIN2*. (d) Sequence analysis of the *TAF1* transcript expressed in *taf1‐2* mutants. ATG codons corresponding to translation AUG start sites in the messenger RNA are indicated in bold. Translation is shown for the ATG codons in frame with wild‐type *TAF1* coding region. The position in corresponding to the SAIL T‐DNA Left Border primer is indicted by the box. An insertion at the T‐DNA/TAF1 border is shown in italics and homology with the T‐DNA is shown in bold. Splice sites are indicated by asterisks.

A *taf1* mutant allele with a T‐DNA insertion in the 5′ UTR (Figure 4a, *taf1‐2*) was identified in the SAIL collection of T‐DNA lines (Sessions *et al*., 2002). Homozygous mutants were viable but displayed growth defects, suggesting that there was sufficient *TAF1* expression in these lines to permit plant growth. Analysis of the insertion present in the *taf1‐2* lines indicated that the left border was inserted towards the 3′ direction of the gene, within the first intron of the 5′ UTR. The T‐DNA construct used in the SAIL lines contains a dual 1′2′ promoter located 96 bases from the left border. This promoter is bidirectional (Velten *et al*., 1984) and in the SAIL T‐DNA, the 1′ portion drives expression of the herbicide resistance selectable marker. The 2′ promoter has previously been reported to drive expression of genes adjacent to the insertion site in SAIL mutant lines (Ülker *et al*., 2008). Analysis of the *taf1‐2* mutants by RT‐PCR confirmed that *TAF1* was expressed in the homozygous mutant lines, and overall transcript levels were similar to those of wild‐type plants (Figure 4b,c) despite mis‐expression from the T‐DNA derived promoter. The *TAF1* mRNA expressed in *taf1‐2* lines includes the T‐DNA left border region (including the region corresponding to the SAIL LB primer) which runs into the *TAF1* coding region, which is correctly spliced in the homozygous mutant line (Figure 4d). Wild‐type *TAF1* has a complex 5′UTR which includes an intron. The spliced *TAF1* mRNA contains two AUGs upstream of the start codon, one of which is out of frame and the other has a stop codon shortly downstream (Figure 4d, nucleotides 258 and 237 respectively). The T‐DNA derived sequence in *taf1‐2* lines adds two additional non‐coding upstream AUG codons to the mature *TAF1* transcript (Figure 4d), which may result in a reduction in translation efficiency.

### Mutant *taf1‐2* lines display pleotropic phenotypes including reduced fertility

**Figure 5:**
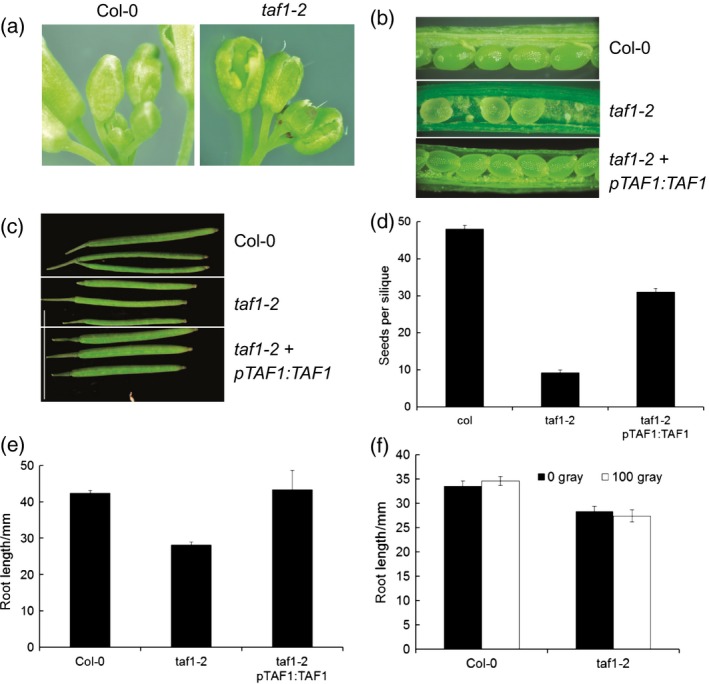
*taf1‐2* mutants display pleotropic effects including reduced fertility. (a) Flowers showing normal flower development (Col‐0) and *taf1‐2* mutant lines with incomplete sepals and purple colouration. (b) High frequency of aborted embryos in *taf1‐2* siliques. (c) *taf1‐2* siliques are a reduced in thickness and slightly reduced in length relative to wild‐type siliques. (d) *taf1‐2* siliques contain reduced numbers of mature seeds. Error bars show SEM of >15 siliques. (e) *taf1‐2* roots are shorter than wild‐type plants but complementation restores wild‐type root growth. Error bars show SEM of >66 roots. (f) *taf1‐2* mutants are not hypersensitive to X‐rays. Error bars show SEM of >35 roots.

Homozygous *taf1‐2* plants demonstrated a number of developmental abnormalities including defective floral morphology. Buds displayed numerous growth defects, with premature opening of the sepals and purple colouration (Figure 5a). *taf1‐2* siliques were significantly smaller and contained a substantial number of aborted embryos, causing a reduction in seed numbers from a mean of 50 seeds per silique in wild‐type to 10 seeds in *taf1‐2* mutant plants. Complementation of *taf1‐2* with the wild‐type *TAF1* gene restored seed numbers to 30 seeds per silique (*P* < 0.01, Figure 5b–d). These phenotypes identify multiple roles for TAF1 in plant floral development and fertility. Homozygous *taf1‐2* also exhibited significantly shorter roots relative to wild‐type plants (*P* < 0.01, Figure 5e), and this phenotype of the mutant lines was fully complemented by transformation with the wild‐type *TAF1* gene (*P* > 0.05). Interaction with the DNA repair factor MRE11 raised the possibility that *taf1‐2* mutant lines may be defective in the response to DNA damage. However, no significant difference was observed in the *taf1‐2* lines in growth relative to wild‐type plants following treatment with X‐rays, which induces single‐ and double‐strand DNA breaks (Figure 5f).

### Loss of the C‐terminal bromodomain does not affect development of *taf1‐3* plants

**Figure 6:**
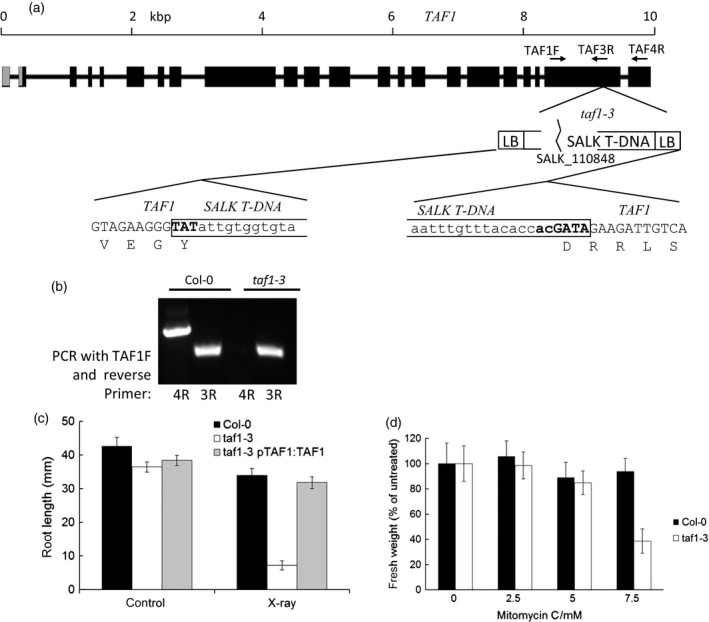
*taf1‐3* mutants are hypersensitive to genotoxic stress. (a) Schematic showing the T‐DNA insertion in *taf1‐3* mutants in exon 20. The translation shows the TAF1 protein sequence. (b) *taf1‐3* plants express a truncated mRNA that lacks the region encoding the C‐terminal bromodomain. (c) *taf1‐3* displays hypersensitivity to X‐rays. Root growth was measured after plating X‐ray treated imbibed seeds on MS plates and grown vertically. Two‐way anova shows a significant interaction between genotype and X‐ray sensitivity for the *taf1‐3* lines (*P* < 0.001) whereas the complemented lines are not significantly different from wild‐type. Error bars show SEM of >15 roots. (d) *taf1‐3* displays hypersensitivity to the interstrand crosslinking reagent mitomycin C (MMC), quantified by measurement of fresh weight after growth in liquid culture supplemented with MMC at the concentrations indicated. Error bars show SEM of >15 roots.

Previous analysis of the *taf1‐3* allele (SALK_110848), in which the T‐DNA is inserted in the 21^st^ exon (Figure 6a), reported that *taf1‐3* (*haf1*) mutants were viable and exhibited normal growth characteristics under standard greenhouse conditions (Bertrand *et al*., 2005). This initial study suggested that *TAF1* showed redundancy with *HAF2/TAF1b* in the essential core transcriptional activities of TFIID (Bertrand *et al*., 2005). The position of the T‐DNA insertion in *taf1‐3* corresponds to the C‐terminal region of the protein (amino acid 1763 of 1919) which would be expected to result in a truncated TAF1 protein lacking the bromodomain (1806–1901), a conserved motif involved in binding acetylated lysine residues (Marchler‐Bauer *et al*., 2011). RT‐PCR and sequence analysis confirmed that homozygous *taf1‐3* lines expressed a truncated mRNA lacking the region encoding the bromodomain (Figure 6b). In agreement with the previous report from Bertrand *et al*. (2005), mutant *taf1‐3* plants show a normal growth phenotype, indistinguishable from wild‐type under normal glasshouse conditions, with normal silique and seed development (Figure S1a,b). Previous reports identified that *haf2/taf1b* mutant plants displayed lighter coloured cotyledons due to a reduced chlorophyll content. In contrast, the *taf1‐3* mutant seedlings were indistinguishable from wild‐type lines, indicating no defects in light regulation of plant development in this line (Figure S1c). However, root growth was reduced in *taf1‐3* plants, which displayed significantly shorter roots than wild‐type lines (*P* < 0.01, Figure 6c). Growth under abiotic stress conditions with either 100 mm NaCl or 200 mm mannitol resulted in a similar reduction in root length at 21 days in both *taf1‐3* and wild‐type lines (*P* > 0.05), indicating no hypersensitivity to salt or osmotic stress in plants lacking the C‐terminal region of TAF1 (Figure S1d). This suggested that stress responses were not impaired in the mutant line and that the truncated mRNA was sufficient for plant growth and development.

### C‐terminal truncation of TAF1 results in hypersensitivity to DNA damage

Identification of specific interaction between TAF1 and MRE11 prompted further investigation into the DNA damage sensitivity of the *taf1‐3* allele. While *taf1‐3* mutants do not display hypersensitivity to abiotic stresses including salt and osmotic stresses (Figure S1d), root growth of homozygous mutants was hypersensitive to X‐ray exposure, with highly significant interaction between genotype and treatment (*P* < 0.001, two‐way factorial anova, Figure 6c). X‐rays induce a range of forms of DNA damage of which DNA DSBs have the greatest biological effect. Significantly, wild‐type levels of X‐ray sensitivity were restored by complementation of the *taf1‐3* line with full‐length *TAF1,* indicating that loss of the C‐terminal bromodomain of the TAF1 protein results in hypersensitivity to genotoxic stress. This was confirmed by the observed hypersensitivity of the *taf1‐3* mutant line to mitomycin C, a bifunctional alkylating agent that crosslinks DNA and inhibits DNA replication (Figure 6d; *P* < 0.01). As growth hypersensitivity was not observed in response to other forms of abiotic stress, including methyl methanesulfonate (MMS) which causes alkylation to bases (Figure S1e), this was indicative of TAF1 playing a role in the plant response to DNA damage and specifically in the forms of DNA damage that require recombinational repair (DSBs and interstrand DNA crosslinks). The transcriptional response to X‐rays, characterised by a high level induction of *RAD51, POLY(ADP)RIBOSE POLYMERASE 2* (*PARP2*), *X‐RAY INDUCED 1* (*XRI1*), *RIBONUCLEOTIDE REDUCTASE* small subunit (*TSO2*) and *THYMIDINE KINASE 1A* (*TK1A*) (Culligan *et al*., 2006; Dean *et al*., 2009) was not significantly different between Col‐0 and *taf1‐3* lines (Figure S1f,g, *P* > 0.05), suggesting that the ATM‐dependent DNA damage response was not impaired in the *taf1‐3* line. These results are consistent with a requirement for the bromodomain‐containing C‐terminal region of TAF1 in the plant DNA damage response, possibly through interaction with MRE11.

### MRE11‐ interaction is mediated by the C‐terminal bromodomain of TAF1

**Figure 7:**
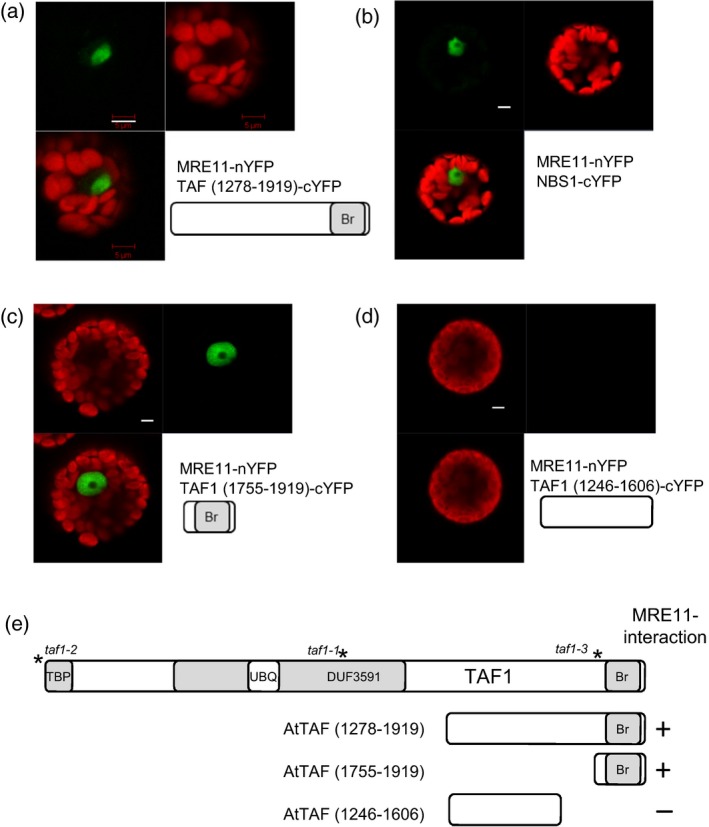
Interaction between MRE11 and the TAF1 bromodomain. (a) Confirmation of MRE11 and TAF1 interaction in planta by split YFP in transient expression in an Arabidopsis protoplast showing nuclear localised TAF1–MRE11 complex (green) and red chlorophyll autofluorescence. (b) Interaction of MRE11–nYFP and NBS1–cYFP. (c) Interaction between the TAF1 bromodomain and MRE11. (d) No interaction observed between MRE11 and the C‐terminal TAF1 region distal to the bromodomain (protoplast showing dsRED fluorescence as a transformation control). (e) Schematic showing the domains of TAF1 tested in BiFC. Scale bars: 5 μm.

To further investigate the roles of the bromodomain region of TAF1, *in planta* interactions between MRE11 and a series of TAF1 deletion constructs were analysed using bimolecular fluorescence complementation. Protein fusions with the C‐ and N‐terminus of the Venus YFP variant were expressed in Arabidopsis leaf protoplasts as described previously (Zhong *et al*., 2008). Initially, the interaction between MRE11 and the C‐terminal region of TAF1 previously identified in the yeast two‐hybrid screen (Figure 1) was investigated. MRE11 was expressed as fusion with the N‐terminal half of YFP (nYFP) and TAF1(1278–1919) was expressed as a fusion with the C‐terminal half of YFP (cYFP). Fluorescence microscopy identified YFP fluorescence localised to the nucleus in transformed protoplasts (Figure 7a). As a positive control, similar evidence of interaction was observed after co‐expression of MRE11‐nYFP with NBS1‐cYFP, confirming previous reports of interaction between these two proteins (Figure 7b) (Waterworth *et al*., 2007). The *in planta* interaction between MRE11 and TAF1 confirmed the results of the yeast two hybrid interaction (Figure 1b), showing interaction between the C‐terminal 641 aa of TAF1 and full‐length MRE11. The role of the bromodomain was further investigated by expression of the C‐terminal 164 aa of TAF1 in BiFC. Interaction between TAF1(1755–1919)‐cYFP and MRE11‐nYFP resulted in YFP fluorescence, indicative of interaction between the bromodomain and MRE11 (Figure 7c). In contrast, interaction was not observed between MRE11‐nYFP and TAF1(1246–1606)‐cYFP which lacked the bromodomain (Figure 7d), or between nYFP alone and the TAF1‐cYFP constructs (Figure S2a,b).

## Discussion

Here we identify physical interaction between the histone acetylase TAF1, a conserved component of the TFIID basal transcription complex, and the recombination endonuclease MRE11, which has important functions in the early stages of chromosomal break repair. Specific hypersensitivity of *taf1‐3* mutants to DNA‐damaging agents that induce DNA crosslinks or DSBs demonstrates a requirement for TAF1 in plant recovery from genotoxic stress. Our studies therefore provide evidence of interaction of a core plant recombination factor, MRE11, with a histone acetylation transferase and thereby identify a potential mechanism for the recruitment of HAT activity to the site of the DNA DSB.

The Arabidopsis genome encodes two TAF isoforms (TAF1 and TAF1b), whereas rice only has one identifiable *TAF1* gene, suggesting possible gene duplication in the Arabidopsis lineage. Diversification has led the TAF1b (HAF2) isoform to evolve specialised physiological functions in the integration of light signalling in Arabidopsis development, activating light‐induced transcription through histone acetyltransferase activity (Benhamed *et al*., 2006). In contrast, TAF1 is essential for plant viability, indicative of a lack of redundancy between the two Arabidopsis *TAF1* genes. The observed lethality of *taf1‐1* homozygous lines is consistent with this being a null allele. It is therefore surprising that the loss of gamete viability in heterozygous lines is not fully penetrant, with around 6% of *taf1‐1* ^*−*^ pollen successfully transmitting the mutant allele. This indicates that *de novo* transcription of the *TAF1* gene is not an absolute requirement for pollen function, reflecting that either residual TAF1 activity is carried over from meiosis or that TAF1‐mediated transcriptional activity is important, but not essential for pollen viability. Similar loss of the mutant allele through the male line has also been observed in Arabidopsis mutants deficient in the TFIID factor AtTAF6 (Lago *et al*., 2005) and in lines deficient in the transcription factor AtTFIIB (Zhou *et al*., 2013), both of which display impaired pollen tube growth. These results indicate that *de novo* transcription is important in pollen growth tube, consistent with the observed dynamic changes in the pollen transcriptome and the sensitivity of germinating Arabidopsis pollen to actinomycin D (Wang *et al*., 2008).

Phenotypic analysis of the hypomorphic *taf1‐2* allele, which expresses a mutant *taf1‐2* transcript driven by the T‐DNA 2′ promoter, revealed a range of pleotropic effects. The mutant transcript contains an extended 5′UTR with multiple non‐coding upstream AUGs which may reduce translation of the full‐length *TAF1* transcript. Plants homozygous for this mutation display defects in root growth, floral development and fertility. The TFIID component TAF10 has also been implicated in floral development. Overexpression of both *Flaveria trinervia TAF10* or *AtTAF10* in Arabidopsis results in floral abnormalities in addition to defects in leaf development (Furumoto *et al*., 2005; Tamada *et al*., 2007). Insertional mutants with reduced AtTAF10 levels displayed shorter roots and a high incidence of arrested vegetative meristems (Tamada *et al*., 2007).

Hypersensitivity of *taf1‐3* to DNA‐damaging reagents that induce crosslinks and DSBs, together with identification of physical association with MRE11, here reveals a role for TAF1 in the plant response to genotoxic stresses. Mammalian cells lacking TAF1 display a constitutively activated DNA damage response, consistent with roles for this histone acetylase in maintaining genome integrity (Buchmann *et al*., 2004). The TFIID transcriptional complex containing TAF1 also binds to different forms of DNA damage including DNA crosslinks, further supporting the association of this factor with mammalian DNA repair (Vichi *et al*., 1997). In plants, an alternative explanation for the DNA damage hypersensitivity observed in the Arabidopsis *taf1‐3* mutant line could be transcriptional dysregulation of the DNA damage response. Whilst it is difficult to completely eliminate this possibility, evidence against this is provided by the normal upregulation of all five genes investigated which represent integral components of the plant transcriptional response to DNA damage. This is indicative that the plant transcriptional response to DNA damage is not eliminated in the *taf1‐3* mutant background. In addition, the mutated transcript expressed in the *taf1‐2* line is sufficient to cause developmental defects but does not result in significant hypersensitivity to DNA damage. This is consistent with the model that the hypersensitivity to DNA damage in *taf1‐3* mutants specifically results from the complete loss of the bromodomain. In this model, the levels of TAF1 that allow survival of *taf1‐2* mutants are also sufficient for wild‐type DNA damage responses in this line. This hypothesis was further supported by complementation of *taf1‐3* with full‐length *TAF1,* which restored wild‐type levels of genotoxin sensitivity to the mutant line. Collectively, these results point to an important role for the C‐terminal bromodomain of TAF1 in the response to damage to the plant genome.

In mammals and yeast, HAT activities are required for efficient recombinational repair of DSBs, and acetylation of histones H3 and H4 plays an important role in survival under genotoxic stress (Bird *et al*., 2002; Tamburini and Tyler, 2005). However, the role of histone acetylation in the plant DSB response and mechanisms is poorly understood. Our previous mass spectroscopy analysis identified a global increase in the relative abundance of the acetylated N‐terminus of H3 and a decrease in H4 acetylation in response to X‐ray‐induced DNA damage in Arabidopsis, indicative of dynamic changes in histone post‐translational modification following genotoxic stress (Drury *et al*., 2012). Acetylation of histone H4 was found on residue K16 together with any combination of K5, K8 and/or K12 acetylation. As in yeast, a significant reduction in H4K16 acetylation is observed in response to DNA damage, which in Arabidopsis requires ATM activity (Tamburini and Tyler, 2005; Drury *et al*., 2012). In response to X‐ray treatment, Arabidopsis histone H3 was hyperacetylated, with modifications detected on K14 with or without K9, and K23 with or without K18 (Drury *et al*., 2012). In yeast, acetylation of residues H3 K14 and K23 have been shown to be critical for responses to DNA damage (Qin and Parthun, 2002). However, the mechanistic basis of histone modification in the plant response to DNA damage remains to be established. In mammals, MRE11 recruits HAT activity to DSBs through interaction with TIP60, which forms part of a complex that participates in DSB repair through acetylation of histones at the site of the break. DSB repair defects caused by TIP60 deficiency can be reversed by chromatin relaxation, suggestive that the TIP60 complex functions to modulate chromatin accessibility at the DSB site (Murr *et al*., 2006). In Arabidopsis, interaction of the C‐terminal region bromodomain of TAF1 with MRE11 provides a potential mechanism for recruitment of HAT activity to a DSB and an explanation for the hypersensitivity of *taf1‐3* mutants specifically to genotoxins.

Our knowledge of the early events in plant DNA damage signalling and repair by recombination pathways remains incomplete despite their crucial importance for cellular survival. Rapid and efficient DNA break recognition is crucial to alleviate the extreme cytotoxic effects of DSBs and the recruitment of MRE11 and associated proteins represents a key step in the initiation of chromosomal break repair. Collectively our studies identify function of TAF1 in the plant response to DNA damage. Further work is required to determine the significance of MRE11‐TAF1 interaction in histone acetylation and the downstream regulation of plant DNA DSB repair pathways. Understanding the mechanisms which control recombination pathways in plants is important in crop resistance to abiotic stress, in meiosis for the generation of new varieties in plant breeding, and in the development of improved gene targeting methodologies.

## Experimental Procedures

### Plant material and growth conditions

*Arabidopsis thaliana* seeds were surface sterilized in 70% ethanol for 5 min, and resuspended in sterile 0.1% agar, stratified at 4°C for 2 days and grown on half‐strength Murashige and Skoog (Sigma, www.sigmaaldrich.com) medium containing 20 g L^*−*1^ sucrose, 0.5 g L^*−*1^ MES pH 5.7, 8 g L^*−*1^ plant agar (Duchefa) for 16 h:8 h light:dark cycles at 22°C. Plants were transferred to soil after 2 weeks and incubated in growth chambers (Sanyo, http://www.panasonic.net/sanyo/) under constant humidity (70%), with 16 h light and 8 h dark cycles at 22°C. Arabidopsis mutant lines were obtained from Nottingham Arabidopsis Stock Centre (Scholl *et al*., 2000). *Agrobacterium*‐mediated plant transformation was performed according to published protocols (Clough and Bent, 1998).

### Nucleic acid purification, amplification and cloning

DNA procedures and bacterial manipulations were by established protocols (Sambrook *et al*., 1989). RNA was isolated from above‐ground tissues of flowering Arabidopsis using the SV total RNA isolation kit (Promega, https://www.promega.com) according to the manufacturer's instructions. Reverse transcriptase (Superscript II, Invitrogen, www.thermofisher.com) was used for cDNA synthesis and PCR amplification for cloning used iProof polymerase (Bio‐Rad, www.Bio-Rad.com) or for analysis used RedTaq (Sigma). DNA extraction for PCR genotyping was performed by grinding plant tissue in shorty buffer (0.2 m Tris pH 9.0, 1% SDS, 0.4 m LiCl, 25 mm EDTA) in a 1.5 ml microfuge tube, using a plastic micropestle. Cell debris was pelleted at 13 000 ***g*** for 5 min and the supernatant mixed 1:1 with 100% isopropanol. Precipitated DNA was recovered by centrifugation at 13 000 ***g*** for 10 min. The dried pellet was resuspended in 400 μl TE buffer. Primers used in plant genotyping were taf1_3_T: CACCGACAGAAAGAGAACAGC; taf1_3_W: AGGTGGTATTCCTGGGTTACG; LBb1_3_SALK: ATTTTGCCGATTTCGGAAC; taf1_1_T: CAATTGCTGCAGATGAGCTGTCTT; taf1_1_W: ACGCAAGTGTGCAACTCCTAGATG; taf1_2_W: CAATCTTGTCTTGGTCGCTTC, taf1_2_T: CAGGCTACAGTAGCCTCCATC; SAIL LB: GCCTTTTCAGAAATGGATAAATAGCCTTGCTTCC. RNA was purified from plant tissue using an SV RNA kit (Promega) and quantified by spectrometry (NanoDrop, www.nanodrop.com). cDNA was synthesised using Invitrogen Superscript II and primed with oligodT. TAF1 cDNA primers were used to determine gene expression and designed to the 20th and 21st exons: TAF1F ACAGAATCACAACCCGAAGG; TAF3R AGGCTTGTGTGATTCGCTCT; TAF4R TCTGGAGCTTCCTTCTTGGA.

### Yeast two‐hybrid analysis

Two‐hybrid analysis was performed as described previously (Fields and Song, 1989; Durfee *et al*., 1993). Full‐length *MRE11* cDNA was cloned into the plasmid pGBKT7 (Clontech, www.clontech.com) in frame with and N‐terminal the GAL4‐DNA binding domain for use in yeast two‐hybrid screening. RNA was isolated from 2‐week‐old Arabidopsis seedlings 2 h after exposure to 10 Gy X‐rays and mixed with an equal quantity of RNA isolated from buds and flowers at various stages of development. RT‐PCR and *in vivo* cloning was used to simultaneously generate an activation domain library and conduct the library screening using the Matchmaker kit according to manufacturer's instructions (Clontech). Transformants were plated directly onto selective media lacking tryptophan, leucine and histidine and supplemented with 2.5 mM 3‐amino‐1,2,4‐triazole. Colonies growing after 1 week were re‐plated onto selective media which also lacked adenine before colony analysis by PCR and sequencing. Interactions were verified by plasmid isolation and re‐transformation into the yeast reporter strain AH109 (MATa, trp1‐901, leu2‐3, 112, ura3‐52, his3‐200, gal4Δ, gal80Δ, LYS2::GAL1UAS‐GAL1TATA‐HIS3, GAL2UAS‐GAL2TATA‐ADE2, URA3::MEL1UAS‐MEL1TATA‐lacZ, MEL1) as described previously (Soni *et al*., 1993).

### Analysis of pollen

DAPI (4′,6‐diamidino‐2‐phenylindole) staining of pollen grains was performed as described by Park *et al*. (2015), and viewed on a Zeiss LSM 510 META Axiovert 200M inverted confocal microscope pollen tube germination was performed according to published procedures (Rodriguez‐Enriquez *et al*., 2013).

### Nucleic acid purification, amplification and cloning

DNA procedures and bacterial manipulations used established protocols (Sambrook *et al*., 1989). RNA was isolated from above‐ground tissues of flowering Arabidopsis using the SV total RNA isolation kit (Promega) according to the manufacturer's instructions. RT‐PCR was performed using Superscript II reverse transcriptase (Invitrogen) for cDNA synthesis followed by amplification using iPROOF (Bio‐Rad). PCR products were cloned using a TOPO‐TA cloning kit and *E. coli* TOP10 cells (Invitrogen), and plasmid DNA was prepared using spin columns (Qiagen, https://www.qiagen.com) prior to DNA sequencing (GATC Biotech, www.gatc-biotech.com). Real‐time PCR analysis was performed on an CFX96 thermocycler (Bio‐Rad) using iQ SYBR Green Supermix (Bio‐Rad) and primers qPCR_ACTf (CTCAGGTATCGCTGACCGTATGAG) and qPCR_ACTr (CTTGGAGATCCACATCTGCTGGAATG) for ACTIN2 (At3g18780). AtRAD51 (At5G20850) was amplified using primers rad51RTf (GTTCTTGAGAAGTCTTCAAGAAGTTAG) and rad51RTr (GCTGAACCATCTACTTGCGCAACTAC). Transcript levels were normalized against those for ACTIN2. Complementation of *taf* mutations was performed using a full‐length genomic clone ligated into pCB1300. TAF1 was amplified using primers TAF1F (GGGTCACTAGTCCGTTGCTGGTTGTTCAAAACTGAC) and TAF1R (GGGTCACTAGTGGGGCCTAAAGAAAGGGTTACA) incorporating *Spe*I sites and ligated into *Xba*I digested pCB1300. Transformed plants were selected on MS medium supplemented with hygromycin (40 mg L^*−*1^) and claforan (50 mg L^*−*1^).

### Bimolecular fluorescence complementation

BiFC was performed as described previously (Zhong *et al*., 2008). MRE11 and TAF1(1278–1919) were amplified and cloned into the entry vector pENTRE (Invitrogen) before subcloning into the split YFP vectors pDH51‐GW‐YFPn and pDH51‐GW‐YFPc. Truncations of TAF1 were made by restriction digestion: the bromodomain‐YFP fusion (TAF1 1755–1919) was constructed using *Xba*I–*Not*I digestion followed by ligation with the filler oligonucleotide GGATATG. The bromodomain was removed (TAF 1246–1606) by *Nsi*I–*Xho*I digestion followed by DNA polymerase fill in (iProof; Bio‐Rad) and ligation. Purified plasmids were used to transform Arabidopsis protoplasts according to the tape‐sandwich method (Wu *et al*., 2009). Fluorescence imaging was performed on a Zeiss (www.zeiss.co.uk) Axiovert 700 inverted confocal microscope.

### Accession numbers

Sequence data from this article can be found in the EMBL/GenBank data libraries under accession number(s) AT1G32750 (TAF1, HAF1), AT3G19040 (TAF1b, HAF2), AT5G54260 (MRE11), At3g02680 (NBS1), At5g20850 (RAD51) and At3g18780 (ACTIN2).

## Supporting information


**Figure S1.** *taf1‐3* mutants are viable and display normal development in the absence of genotoxic stress.

**Figure S2.** Controls for the interaction between MRE11 and the TAF1 bromodomain.

**Table S1.** PCR primers used in this study.

## References

[tpj13020-bib-0001] Alonso, J.M. , Stepanova, A.N. , Leisse, T.J. ***et al.*** (2003) Genome‐wide insertional mutagenesis of *Arabidopsis thaliana* . Science, 301, 653–657. 12893945 10.1126/science.1086391

[tpj13020-bib-0002] Benhamed, M. , Bertrand, C. , Servet, C. and Zhou, D.X. (2006) Arabidopsis GCN5, HD1, and TAF1/HAF2 interact to regulate histone acetylation required for light‐responsive gene expression. Plant Cell, 18, 2893–2903. 17085686 10.1105/tpc.106.043489 PMC1693931

[tpj13020-bib-0003] Bertrand, C. , Benhamed, M. , Li, Y.‐F. , Ayadi, M. , Lemonnier, G. , Renou, J.‐P. , Delarue, M. and Zhou, D.‐X. (2005) Arabidopsis HAF2 gene encoding TATA‐binding protein (TBP)‐associated factor TAF1, is required to integrate light signals to regulate gene expression and growth. J. Biol. Chem. 280, 1465–1473. 15525647 10.1074/jbc.M409000200

[tpj13020-bib-0004] Bird, A.W. , Yu, D.Y. , Pray‐Grant, M.G. , Qiu, Q. , Harmon, K.E. , Megee, P.C. , Grant, P.A. , Smith, M.M. and Christman, M.F. (2002) Acetylation of histone H4 by Esa1 is required for DNA double‐strand break repair. Nature, 419, 411. 12353039 10.1038/nature01035

[tpj13020-bib-0005] Buchmann, A.M. , Skaar, J.R. and DeCaprio, J.A. (2004) Activation of a DNA damage checkpoint response in a TAF1‐defective cell line. Mol. Cell. Biol. 24, 5332–5339. 15169897 10.1128/MCB.24.12.5332-5339.2004 PMC419897

[tpj13020-bib-0006] Bundock, P. and Hooykaas, P. (2002) Severe developmental defects, hypersensitivity to DNA‐damaging agents, and lengthened telomeres in Arabidopsis MRE11 mutants. Plant Cell, 14, 2451–2462. 12368497 10.1105/tpc.005959 PMC151228

[tpj13020-bib-0007] Clough, S.J. and Bent, A.F. (1998) Floral dip: a simplified method for Agrobacterium‐mediated transformation of *Arabidopsis thaliana* . Plant J. 16, 735–743. 10069079 10.1046/j.1365-313x.1998.00343.x

[tpj13020-bib-0008] Culligan, K.M. , Robertson, C.E. , Foreman, J. , Doerner, P. and Britt, A.B. (2006) ATR and ATM play both distinct and additive roles in response to ionizing radiation. Plant J. 48, 947–961. 17227549 10.1111/j.1365-313X.2006.02931.x

[tpj13020-bib-0009] De Muyt, A. , Pereira, L. , Vezon, D. ***et al.*** (2009) A high throughput genetic screen identifies new early meiotic recombination functions in *Arabidopsis thaliana* . PLoS Genet. 5, e1000654. 19763177 10.1371/journal.pgen.1000654 PMC2735182

[tpj13020-bib-0010] Dean, P.J. , Siwiec, T. , Waterworth, W.M. , Schlogelhofer, P. , Armstrong, S.J. and West, C.E. (2009) A novel ATM‐dependent X‐ray‐inducible gene is essential for both plant meiosis and gametogenesis. Plant J. 58, 791–802. 19187040 10.1111/j.1365-313X.2009.03814.x PMC4143975

[tpj13020-bib-0011] Drury, G.E. , Dowle, A.A. , Ashford, D.A. , Waterworth, W.M. , Thomas, J. and West, C.E. (2012) Dynamics of plant histone modifications in response to DNA damage. Biochem. J. 445, 393–401. 22574698 10.1042/BJ20111956

[tpj13020-bib-0012] Durfee, T. , Becherer, K. , Chen, P.L. , Yeh, S.H. , Yang, Y. , Kilburn, A.E. , Lee, W.H. and Elledge, S.J. (1993) The retinoblastoma protein associates with the protein phosphatase type 1 catalytic subunit. Genes Dev. 7, 555–569. 8384581 10.1101/gad.7.4.555

[tpj13020-bib-0013] Earley, K.W. , Shook, M.S. , Brower‐Toland, B. , Hicks, L. and Pikaard, C.S. (2007) *In vitro* specificities of Arabidopsis co‐activator histone acetyltransferases: implications for histone hyperacetylation in gene activation. Plant J. 52, 615–626. 17877703 10.1111/j.1365-313X.2007.03264.x

[tpj13020-bib-0014] Falck, J. , Coates, J. and Jackson, S.P. (2005) Conserved modes of recruitment of ATM, ATR and DNA‐PKcs to sites of DNA damage. Nature, 434, 605–611. 15758953 10.1038/nature03442

[tpj13020-bib-0015] Fields, S. and Song, O. (1989) A novel genetic system to detect protein‐protein interactions. Nature, 340, 245–246. 2547163 10.1038/340245a0

[tpj13020-bib-0016] Furumoto, T. , Tamada, Y. , Izumida, A. , Nakatani, H. , Hata, S. and Izui, K. (2005) Abundant expression in vascular tissue of plant TAF10, an orthologous gene for TATA box‐binding protein‐associated factor 10, in *Flaveria trinervia* and abnormal morphology of *Arabidopsis thaliana* transformants on its overexpression. Plant Cell Physiol. 46, 108–117. 15659449 10.1093/pcp/pci006

[tpj13020-bib-0017] Heacock, M. , Spangler, E. , Riha, K. , Puizina, J. and Shippen, D.E. (2004) Molecular analysis of telomere fusions in Arabidopsis: multiple pathways for chromosome end‐joining. EMBO J. 23, 2304–2313. 15141167 10.1038/sj.emboj.7600236 PMC419913

[tpj13020-bib-0018] Kanno, T. , Kanno, Y. , Siegel, R.M. , Jang, M.K. , Lenardo, M.J. and Ozato, K. (2004) Selective recognition of acetylated histones by bromodomain proteins visualized in living cells. Mol. Cell, 13, 33–43. 14731392 10.1016/s1097-2765(03)00482-9

[tpj13020-bib-0019] Lago, C. , Clerici, E. , Mizzi, L. , Colombo, L. and Kater, M.M. (2004) TBP‐associated factors in Arabidopsis. Gene, 342, 231–241. 15527982 10.1016/j.gene.2004.08.023

[tpj13020-bib-0020] Lago, C. , Clerici, E. , Dreni, L. , Horlow, C. , Caporali, E. , Colombo, L. and Kater, M.M. (2005) The Arabidopsis TFIID factor AtTAF6 controls pollen tube growth. Dev. Biol. 285, 91–100. 16039640 10.1016/j.ydbio.2005.06.006

[tpj13020-bib-0021] Lawit, S. , O'Grady, K. , Gurley, W. and Czarnecka‐Verner, E. (2007) Yeast two‐hybrid map of Arabidopsis TFIID. Plant Mol. Biol. 64, 73–87. 17340043 10.1007/s11103-007-9135-1

[tpj13020-bib-0022] Maile, T. , Kwoczynski, S. , Katzenberger, R.J. , Wassarman, D.A. and Sauer, F. (2004) TAF1 activates transcription by phosphorylation of serine 33 in histone H2B. Science, 304, 1010–1014. 15143281 10.1126/science.1095001

[tpj13020-bib-0023] Marchler‐Bauer, A. , Lu, S. , Anderson, J.B. ***et al.*** (2011) CDD: a conserved domain database for the functional annotation of proteins. Nucleic Acids Res. 39, D225–D229. 21109532 10.1093/nar/gkq1189 PMC3013737

[tpj13020-bib-0024] Martinez, E. (2002) Multi‐protein complexes in eukaryotic gene transcription. Plant Mol. Biol. 50, 925–947. 12516863 10.1023/a:1021258713850

[tpj13020-bib-0025] Mizzen, C.A. , Yang, X.‐J. , Kokubo, T. ***et al.*** (1996) The TAFII250 subunit of TFIID has histone acetyltransferase activity. Cell, 87, 1261–1270. 8980232 10.1016/s0092-8674(00)81821-8

[tpj13020-bib-0026] Murr, R. , Loizou, J.I. , Yang, Y.G. , Cuenin, C. , Li, H. , Wang, Z.Q. and Herceg, Z. (2006) Histone acetylation by Trrap‐Tip60 modulates loading of repair proteins and repair of DNA double‐strand breaks. Nat. Cell Biol. 8, 91–99. 16341205 10.1038/ncb1343

[tpj13020-bib-0027] Pandey, R. , Muller, A. , Napoli, C.A. , Selinger, D.A. , Pikaard, C.S. , Richards, E.J. , Bender, J. , Mount, D.W. and Jorgensen, R.A. (2002) Analysis of histone acetyltransferase and histone deacetylase families of *Arabidopsis thaliana* suggests functional diversification of chromatin modification among multicellular eukaryotes. Nucleic Acids Res. 30, 5036–5055. 12466527 10.1093/nar/gkf660 PMC137973

[tpj13020-bib-0800] Park, S.Y. , Vaghchhipawala, Z. , Vasudevan, B. *et al* (2015) Agrobacterium T‐DNA integration into the plant genome can occur without the activity of key non‐homologous end‐joining proteins. Plant J, 81, 934–946. 25641249 10.1111/tpj.12779

[tpj13020-bib-0028] Pham, A.D. and Sauer, F. (2000) Ubiquitin‐activating/conjugating activity of TAFII250, a mediator of activation of gene expression in Drosophila. Science, 289, 2357–2360. 11009423 10.1126/science.289.5488.2357

[tpj13020-bib-0029] Puizina, J. , Siroky, J. , Mokros, P. , Schweizer, D. and Riha, K. (2004) Mre11 deficiency in Arabidopsis is associated with chromosomal instability in somatic cells and Spo11‐dependent genome fragmentation during meiosis. Plant Cell, 16, 1968–1978. 15258261 10.1105/tpc.104.022749 PMC519189

[tpj13020-bib-0030] Qin, S. and Parthun, M.R. (2002) Histone H3 and the histone acetyltransferase Hat1p contribute to DNA double‐strand break repair. Mol. Cell. Biol. 22, 8353–8365. 12417736 10.1128/MCB.22.23.8353-8365.2002 PMC134061

[tpj13020-bib-0031] Raut, V.V. and Sainis, J.K. (2011) (60) Co‐gamma radiation induces differential acetylation and phosphorylation of histones H3 and H4 in wheat. Plant Biol. 14, 110–117. 21973290 10.1111/j.1438-8677.2011.00463.x

[tpj13020-bib-0032] Rodriguez‐Enriquez, M.J. , Mehdi, S. , Dickinson, H.G. and Grant‐Downton, R.T. (2013) A novel method for efficient *in vitro* germination and tube growth of *Arabidopsis thaliana* pollen. New Phytol. 197, 668–679. 23173941 10.1111/nph.12037

[tpj13020-bib-0033] Sambrook, J. , Fritsch, E.F. and Manniatis, T. (1989) Molecular Cloning: A Laboratory Manual. Cold Spring Harbor, NY: Cold Spring Harbor Press.

[tpj13020-bib-0034] Scholl, R.L. , May, S.T. and Ware, D.H. (2000) Seed and molecular resources for Arabidopsis. Plant Physiol. 124, 1477–1480. 11115863 10.1104/pp.124.4.1477 PMC1539300

[tpj13020-bib-0035] Sessions, A. , Burke, E. , Presting, G. ***et al.*** (2002) A high‐throughput Arabidopsis reverse genetics system. Plant Cell, 14, 2985–2994. 12468722 10.1105/tpc.004630 PMC151197

[tpj13020-bib-0036] Soni, R. , Carmichael, J.P. and Murray, J.A. (1993) Parameters affecting lithium acetate‐mediated transformation of *Saccharomyces cerevisiae* and development of a rapid and simplified procedure. Curr. Genet. 24, 455–459. 8299163 10.1007/BF00351857

[tpj13020-bib-0037] Tamada, Y. , Nakamori, K. , Nakatani, H. , Matsuda, K. , Hata, S. , Furumoto, T. and Izui, K. (2007) Temporary expression of the TAF10 gene and its requirement for normal development of *Arabidopsis thaliana* . Plant Cell Physiol. 48, 134–146. 17148695 10.1093/pcp/pcl048

[tpj13020-bib-0038] Tamburini, B.A. and Tyler, J.K. (2005) Localized histone acetylation and deacetylation triggered by the homologous recombination pathway of double‐strand DNA repair. Mol. Cell. Biol. 25, 4903–4913. 15923609 10.1128/MCB.25.12.4903-4913.2005 PMC1140608

[tpj13020-bib-0039] Ülker, B. , Peiter, E. , Dixon, D.P. , Moffat, C. , Capper, R. , Bouché, N. , Edwards, R. , Sanders, D. , Knight, H. and Knight, M.R. (2008) Getting the most out of publicly available T‐DNA insertion lines. Plant J. 56, 665–677. 18644000 10.1111/j.1365-313X.2008.03608.x

[tpj13020-bib-0040] Velten, J. , Velten, L. , Hain, R. and Schell, J. (1984) Isolation of a dual plant promoter fragment from the Ti plasmid of *Agrobacterium tumefaciens* . EMBO J. 3, 2723–2730. 16453574 10.1002/j.1460-2075.1984.tb02202.x PMC557759

[tpj13020-bib-0041] Vichi, P. , Coin, F. , Renaud, J.P. , Vermeulen, W. , Hoeijmakers, J.H.J. , Moras, D. and Egly, J.M. (1997) Cisplatin‐ and UV‐damaged DNA lure the basal transcription factor TFIID/TBP. 10.1093/emboj/16.24.7444 PMC1170344 9405373

[tpj13020-bib-0042] Wang, Y. , Zhang, W.‐Z. , Song, L.‐F. , Zou, J.‐J. , Su, Z. and Wu, W.‐H. (2008) Transcriptome analyses show changes in gene expression to accompany pollen germination and tube growth in Arabidopsis. Plant Physiol. 148, 1201–1211. 18775970 10.1104/pp.108.126375 PMC2577266

[tpj13020-bib-0043] Wassarman, D.A. and Sauer, F. (2001) TAFII250. J. Cell Sci. 114, 2895–2902. 11686293 10.1242/jcs.114.16.2895

[tpj13020-bib-0044] Waterworth, W.M. , Altun, C. , Armstrong, S.J. , Roberts, N. , Dean, P.J. , Young, K. , Weil, C.F. , Bray, C.M. and West, C.E. (2007) NBS1 is involved in DNA repair and plays a synergistic role with ATM in mediating meiotic homologous recombination in plants. Plant J. 52, 41–52. 17672843 10.1111/j.1365-313X.2007.03220.x

[tpj13020-bib-0045] Waterworth, W.M. , Drury, G.E. , Bray, C.M. and West, C.E. (2011) Repairing breaks in the plant genome: the importance of keeping it together. New Phytol. 192, 805–822. 21988671 10.1111/j.1469-8137.2011.03926.x

[tpj13020-bib-0046] Wu, F.H. , Shen, S.C. , Lee, L.Y. , Lee, S.H. , Chan, M.T. and Lin, C.S. (2009) Tape‐Arabidopsis Sandwich – a simpler Arabidopsis protoplast isolation method. Plant Methods, 5, 16. 19930690 10.1186/1746-4811-5-16 PMC2794253

[tpj13020-bib-0047] Zhong, S. , Lin, Z. , Fray, R.G. and Grierson, D. (2008) Improved plant transformation vectors for fluorescent protein tagging. Transgenic Res. 17, 985–989. 18594998 10.1007/s11248-008-9199-y PMC2522295

[tpj13020-bib-0048] Zhou, J.J. , Liang, Y. , Niu, Q.K. , Chen, L.Q. , Zhang, X.Q. and Ye, D. (2013) The Arabidopsis general transcription factor TFIIB1 (AtTFIIB1) is required for pollen tube growth and endosperm development. J. Exp. Bot. 64, 2205–2218. 23547107 10.1093/jxb/ert078 PMC3654413

