# Supplementary material for: Arabidopsis TAF1 is an MRE11‐interacting protein required for resistance to genotoxic stress and viability of the male gametophyte
Source: Plant J. 2015 Oct 8;84(3):545–57. doi: 10.1111/tpj.13020 (PMC4949998; doi:10.1111/tpj.13020)
Supplement: Supplementary file 2 — Figure S2. Controls for the interaction between MRE11 and the TAF1 bromodomain. [file TPJ-84-545-s002.pdf]

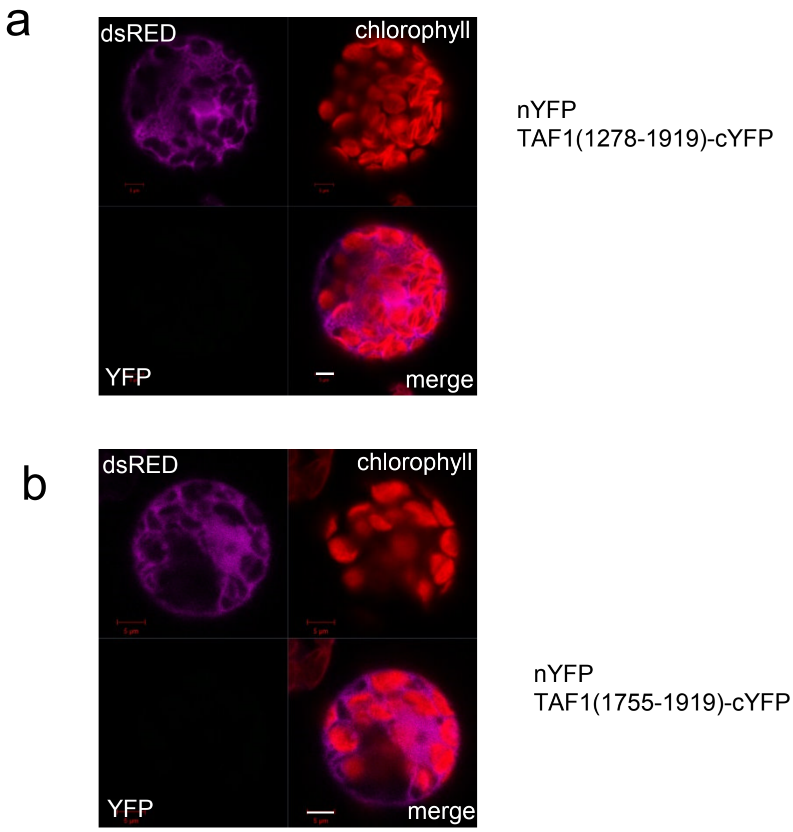

Supporting data Figure 2: Controls for the interaction between MRE11 and the TAF1 bromodomain. (a) Transient expression in an Arabidopsis protoplast showing red chlorophyll autofluorescence. TAF1(1278-1919)-cYFP does not interact with untagged nYFP. dsRED provides a transformation control (magenta) (b) TAF1(1755-1919)-cYFP does not interact with untagged nYFP. dsRED provides a transformation control (magenta). Scale bar is 5μm.
