# Supplementary material for: Arabidopsis TAF1 is an MRE11‐interacting protein required for resistance to genotoxic stress and viability of the male gametophyte
Source: Plant J. 2015 Oct 8;84(3):545–57. doi: 10.1111/tpj.13020 (PMC4949998; doi:10.1111/tpj.13020)
Supplement: Supplementary file 3 — Table S1. PCR primers used in this study. [file TPJ-84-545-s003.pdf]

Supporting data Table 1: PCR primers used in this study

ACTIN2 (At3g18780)	qPCR_ACTf CTCAGGTATCGCTGACCGTATGAG)	qPCR_ACTr CTTGGAGATCCACATCTGCTGGAATG
AtRAD51 (At5G20850)	rad51RTf GTTCTTGAGAAGTCTTCAAGAAGTTAG	rad51RTr GCTGAACCATCTACTTGCGCAACTAC
AtTK1A (AT3G07800)	TK1A_f TGATCGTTGCTGGCCTAGAT	TK1A_r AGCTCAGGTCTGGTGTCACA
TSO2 (AT3G27060)	TSO2R GCTAGACATAACGGAAGCCTTT	TSO2F GTTTGTTGCGGATAGGCTTTTG
XRI1 (AT5G48720)	XRI F GCTACCTGATGACTTAAACTTTGGTTC	XRI R CATTTGGAGAAGATCGAGTCACAG
AtPARP2 (AT4G02390)	PARP2F GCAGCGGTGTTAGATCAGTG	PARP2R CCAACTCTTCCCCATCTGGT
TAF1 genomic clone	TAF1F GGGTCACTAGTCCGTTGCTGGTTGTTCAAAACTGAC	TAF1R GGGTCACTAGTGGGGCCTAAAGAAAGGGTTACA
taf1-3 WT allele	taf1_3_T: CACCGACAGAAAGAGAACAGC	taf1_3_W: AGGTGGTATTCCTGGGTTACG
taf1_3 mutant allele	taf1_3_T: CACCGACAGAAAGAGAACAGC	LBb1_3_SALK: ATTTTGCCGATTTCGGAAC
taf1_1 WT allele	taf1_1_T: CAATTGCTGCAGATGAGCTGTCTT	taf1_1_W: ACGCAAGTGTGCAACTCCTAGATG
taf1_1 mutant allele	taf1_1_T: CAATTGCTGCAGATGAGCTGTCTT	LBb1_3_SALK: ATTTTGCCGATTTCGGAAC
taf1_2 WT allele	taf1_2_W: CAATCTTGTCTTGGTCGCTTC	taf1_2_T: CAGGCTACAGTAGCCTCCATC
taf1_2 mutant allele	taf1_2_T: CAGGCTACAGTAGCCTCCATC	SAIL LB: GCC TTT TCA GAA ATG GAT AAA TAG CCT TGC TTC C
TAF1 exon 20	TAF1F ACAGAATCACAACCCGAAGG	TAF3R AGGCTTGTGTGATTCGCTCT
TAF1 exon 20-21	TAF1F ACAGAATCACAACCCGAAGG	TAF4R TCTGGAGCTTCCTTCTTGGA
