# Supplementary material for: Arabidopsis TAF1 is an MRE11‐interacting protein required for resistance to genotoxic stress and viability of the male gametophyte
Source: Plant J. 2015 Oct 8;84(3):545–57. doi: 10.1111/tpj.13020 (PMC4949998; doi:10.1111/tpj.13020)
Supplement: Supplementary file 4 [file TPJ-84-545-s004.docx]

**Supporting data Figure 1: *taf1-3* mutants are viable and display normal development in the absence of genotoxic stress.** (a) *taf1-3* display no increase in seed abortion relative to Col-0 (b) *taf1-3* siliques are similar to wild type. Bar is 1cm. (c) Light responses are normal in *taf1-3* in contrast to *taf1b* (*haf2*) mutants (Bertrand et al., 2008) (d) Root growth in wild type and *taf1-3* mutants after treatment with abiotic stresses including salt (NaCl) and osmotic stress (mannitol). (e) Root growth in wild type and *taf1-3* mutants after treatment with MMS. (f) There is no significant difference in the DNA damage responsive transcriptional induction of *AtRAD51* (AT5G20850) in *taf1-3* mutants and wild type lines. Values are normalised to *ACTIN2*. (g) Transcriptional induction of DNA damage responsive genes *PARP2* (AT4G02390), *XRI* (AT5G48720), *RNR* (AT3G27060) and *TK1A* (AT3G07800) normalised to *ACTIN2* and relative to untreated Col-0.

**Supporting data Figure 2: Controls for the interaction between MRE11 and the TAF1 bromodomain.** (a) Transient expression in an Arabidopsis protoplast showing red chlorophyll autofluorescence. TAF1(1278-1919)-cYFP does not interact with untagged nYFP. dsRED provides a transformation control (magenta) (b) TAF1(1755-1919)-cYFP does not interact with untagged nYFP. dsRED provides a transformation control (magenta). Scale bar is 5μm.

**Supporting Data Table 1:** PCR primers used in this study.
